# Supplementary figures and images for: Admixture mapping identifies genetic regions associated with blood pressure phenotypes in African Americans
Source: PLoS One. 2020 Apr 21;15(4):e0232048. doi: 10.1371/journal.pone.0232048 (PMC7173845; doi:10.1371/journal.pone.0232048)

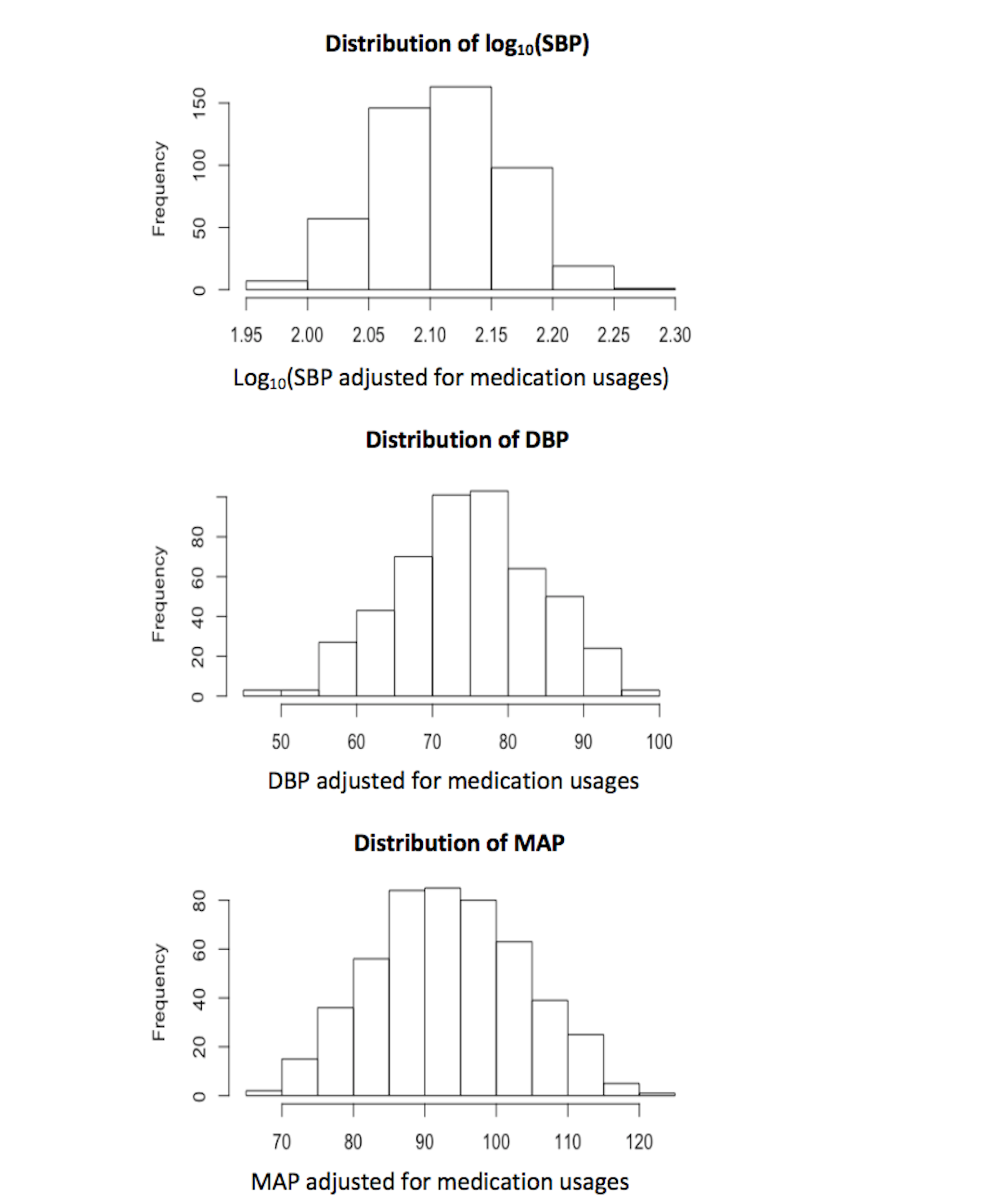

Supplement: S1 Fig — The phenotype distributions are approximately normal. (TIFF) [file pone.0232048.s001.tiff]

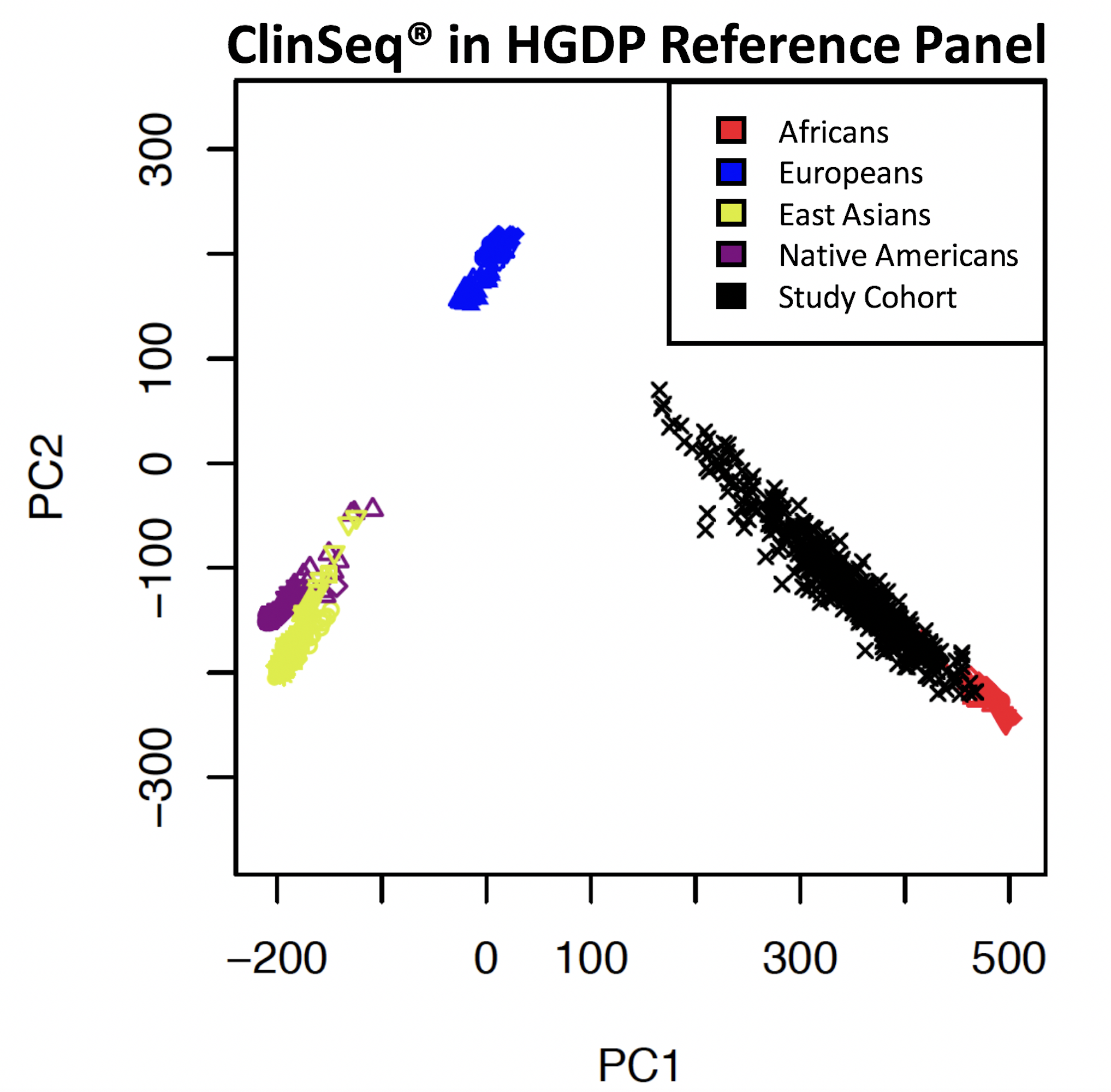

Supplement: S2 Fig — This figure shows principal components 1 and 2 of the ClinSeq® A2 dataset using the HGDP reference panel. The red cluster represents African ancestral populations; the blue cluster represents European ancestral populations; the yellow cluster represents East Asian populations, and the purple cluster represents Native American populations from the HGDP reference panel. The black cluster represents the ClinSeq® study. (TIFF) [file pone.0232048.s002.tiff]

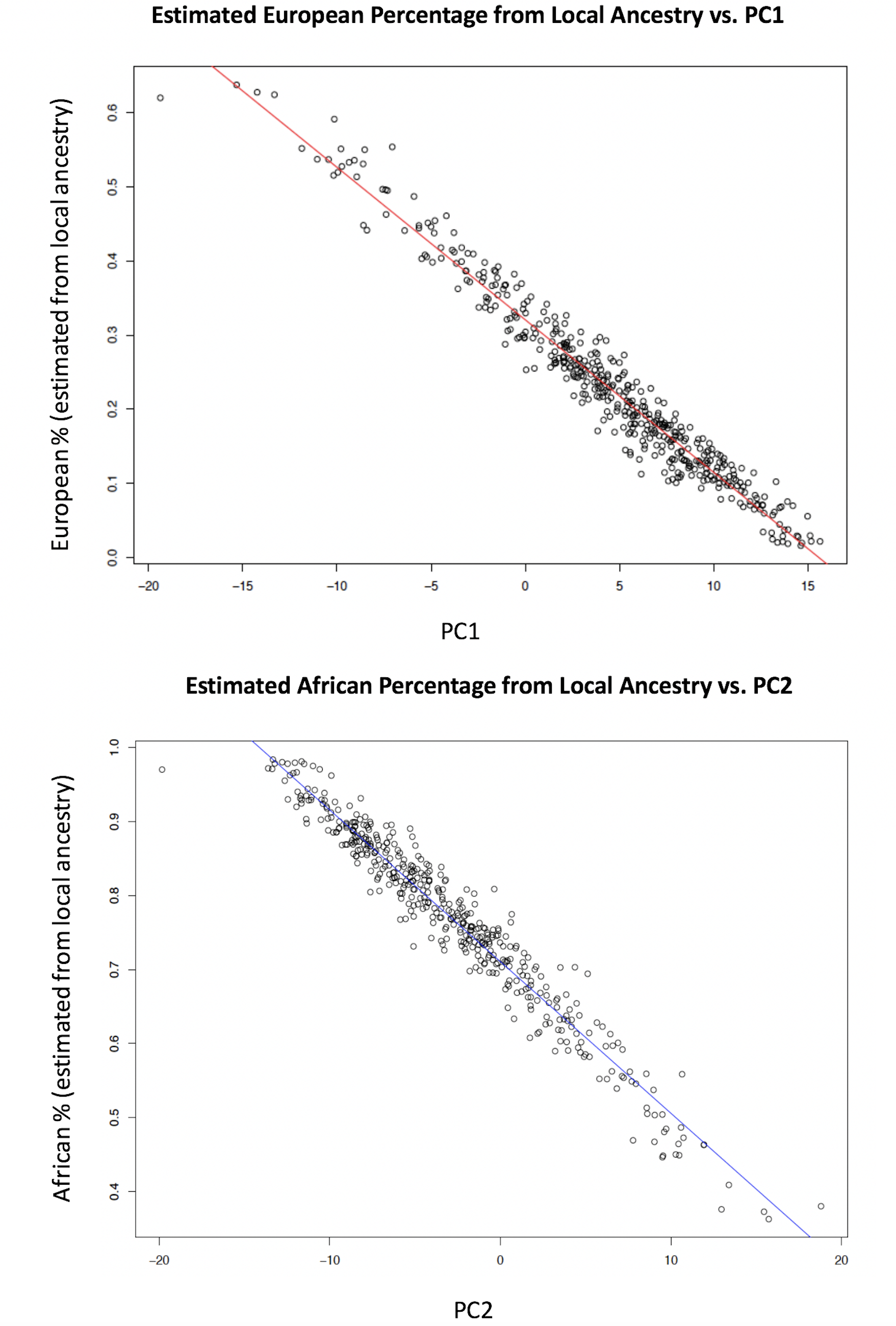

Supplement: S3 Fig — This figure shows the global European ancestry estimated by averaging local ancestry across the entire exome plotted against principal component 1 as estimated by LASER, and the global African ancestry plotted against principal component 2. The X axis denotes global ancestry; the Y axis denotes principal components. Each dot denotes an African American individual who has passed QC. (TIFF) [file pone.0232048.s003.tiff]

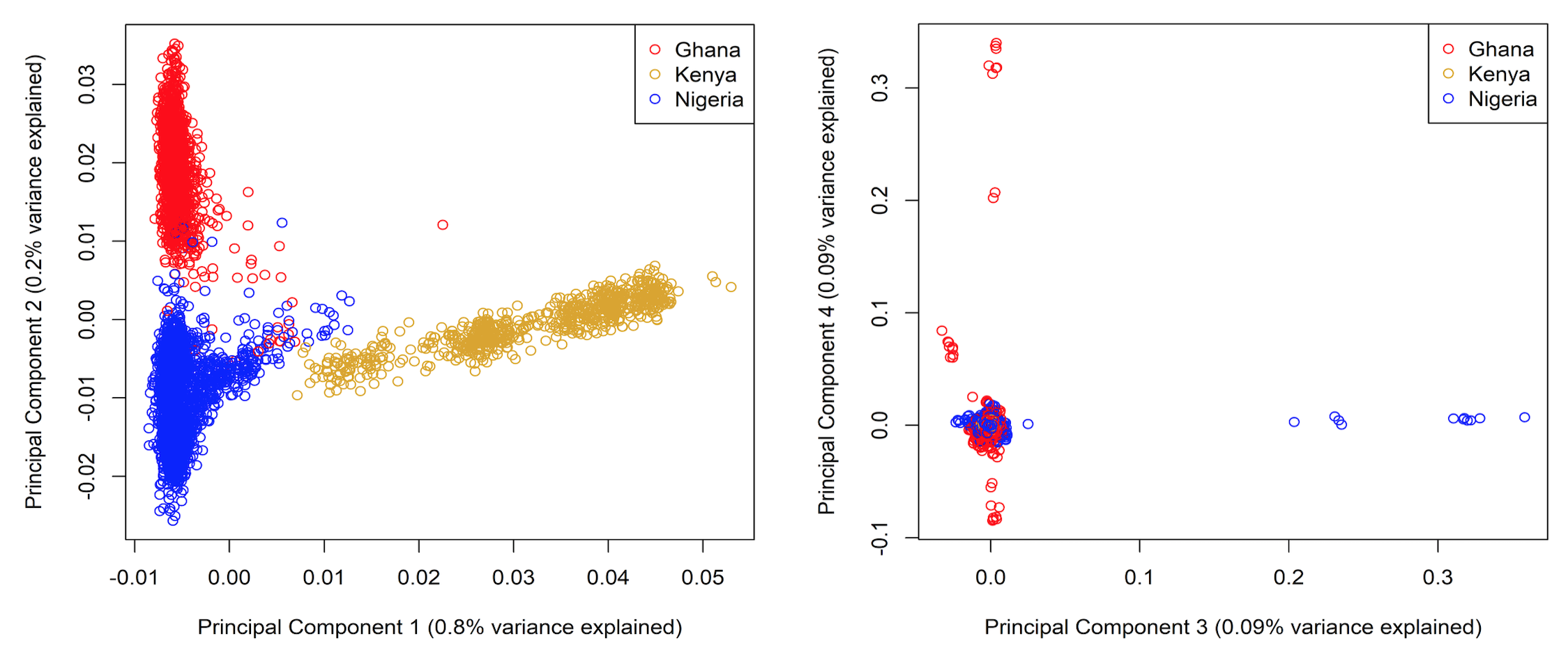

Supplement: S4 Fig — Principal component 1 separates Kenyans from Ghanaians and Nigerians and also separates the Kenyans. Principal component 2 separates Ghanaians from Nigerians. Principal component 3 separates 11 Yoruba. (TIFF) [file pone.0232048.s004.tiff]
