# Supplementary material for: Admixture mapping identifies genetic regions associated with blood pressure phenotypes in African Americans
Source: PLoS One. 2020 Apr 21;15(4):e0232048. doi: 10.1371/journal.pone.0232048 (PMC7173845; doi:10.1371/journal.pone.0232048)
Supplement: S1 Table — (DOCX) [file pone.0232048.s005.docx]

**S1 Table.** **Replication study in the AADM dataset.**

| **Chr** | **BP** | **rsID** | **A1** | **A2** | **ClinSeq®** | | | **AADM** | | | | |  |  |  |
| --- | --- | --- | --- | --- | --- | --- | --- | --- | --- | --- | --- | --- | --- | --- | --- |
|  |  |  |  |  | **Freq_A1_** | **Beta** | ***P*-value** | **Freq_A1_** | **Beta** | | ***P*-value^a^** | |  |  |  |
| **DBP** |  |  |  |  |  |  |  |  |  | |  | |  |  |  |
| 20 | 2597978 | rs4815428 | G | A | 0.75 | -2.42 | 9.57E-04 | 0.86 | 0.03 | | 0.40 | |  |  |  |
| **MAP** |  |  |  |  |  |  |  |  |  | |  | |  |  |  |
| 1 | 150975108 | rs771205 | T | C | 0.57 | -1.99 | 3.37E-03 | 0.65 | -0.04 | | 0.18 | |  |  |  |
| 1 | 152276889 | rs3126067 | G | A | 0.70 | -2.37 | 1.39E-03 | 0.79 | 0.05 | | 0.11 | |  |  |  |
| 1 | 152280782 | rs2184953 | G | A | 0.70 | -2.33 | 1.44E-03 | 0.79 | 0.06 | | 0.08 | |  |  |  |
| 1 | 152283862 | rs58001094 | C | G | 0.70 | -2.33 | 1.44E-03 | 0.78 | 0.06 | | 0.07 | |  |  |  |
| ^a^ Multiple testing thresholds were 0.05 for DBP and 0.05/4 = 0.0125 for MAP in AADM. | | | | | | | | | |  | |  | |  |  |
