# Supplementary material for: Admixture mapping identifies genetic regions associated with blood pressure phenotypes in African Americans
Source: PLoS One. 2020 Apr 21;15(4):e0232048. doi: 10.1371/journal.pone.0232048 (PMC7173845; doi:10.1371/journal.pone.0232048)
Supplement: S2 Table — (DOCX) [file pone.0232048.s006.docx]

**S2 Table. Meta-analyses of local-ancestry stratified genotypic association tests.**

| **Chr** | **BP** | **rsID** | **No. of strata** | **Beta** | **Std. error** | ***P*-value^a^** |
| --- | --- | --- | --- | --- | --- | --- |
| **DBP** |  |  |  |  |  |  |
| 20 | 2597978 | rs4815428 | 3 | -2.10 | 0.84 | 0.01 |
| **MAP** |  |  |  |  |  |  |
| 1 | 150975108 | rs771205 | 3 | -0.90 | 1.79 | 0.61 |
| 1 | 152276889 | rs3126067 | 3 | -1.62 | 1.46 | 0.27 |
| 1 | 152280782 | rs2184953 | 3 | -1.63 | 1.42 | 0.25 |
| 1 | 152283862 | rs58001094 | 3 | -1.63 | 1.42 | 0.25 |
| ^a^ Each SNP is an independent test and the significant threshold is 0.05. | | | | | | |
